# Thyroid eye disease or Graves’ orbitopathy: What name to use, and why it matters

**DOI:** 10.3389/fendo.2022.1083886

**Published:** 2022-11-28

**Authors:** Lilly H. Wagner, Elizabeth A. Bradley, Andrea A. Tooley, Yanhan Ren, Kharisa N. Rachmasari, Marius N. Stan

**Affiliations:** ^1^ Department of Ophthalmology, Mayo Clinic, Rochester, MN, United States; ^2^ Division of Endocrinology, Mayo Clinic, Rochester, MI, United States

**Keywords:** thyroid eye disease, graves, orbitopathy, ophthalmopathy, TED

## Abstract

There is currently no universally accepted name for inflammatory disease of the eye and orbit associated with thyroid autoimmune disease. Variability in terminology impedes the evaluation of scientific literature and clinical collaboration and can affect patients’ understanding of a disease process. The goals of this perspective article are 1. To compare the frequency of different terms used for eye disease associated with autoimmune thyroid disease in the scientific literature between 2000, 2010 and 2020 publications; 2. To investigate potential associations of terminology with author and journal specialty, and multidisciplinary vs. mono-disciplinary author teams; 3. To determine preferential terms used by professional societies; and 4. To propose standardized terminology based on our data analysis. The methods for this study included review of all English language articles listed in PubMed, with publication dates in the years 2000, 2010 and 2020, that included one of 6 terms currently used to describe eye disease associated with autoimmune thyroid disease. Characteristics pertaining to authors, journals, and article type were recorded. Results showed that the most used term in the 2000 literature was Graves’ Ophthalmopathy (61%). In the 2010 literature, Graves’ Orbitopathy (31%) became most common, followed by Graves’ Ophthalmopathy (30%). Between 2010 and 2020, thyroid eye disease (37%) became the most common term, followed by Graves’ Orbitopathy (35%). This perspective article proposes “thyroid eye disease” (TED) as the preferred name for this entity and discusses supporting terminology patterns and trends over time in scientific literature and in professional societies.

## Introduction

Standardization of medical terminology is crucial to facilitate the evaluation of scientific literature, ensure accuracy of diagnostic parameters and reported outcomes, and allow for multi-center collaboration. In addition, patients’ understanding of a disease process and treatment decisions can be impacted by terminology changes (1). Results of standardization efforts are frequently published as practice guidelines or consensus reports by large specialty groups (2, 3). There are little published data on terminology for inflammatory disease of the eye and orbit associated with thyroid autoimmune disease. Currently used terms include thyroid eye disease (TED), Graves’ orbitopathy, thyroid-associated orbitopathy, Graves’ ophthalmopathy, Graves eye disease and Basedow disease.

Ideally, terminology for a medical condition appropriately describes the involved anatomical structures and disease process, allows clinicians to differentiate the diagnosis from similar entities, and can be understood by patients and other specialties alike. This perspective article summarizes terminology patterns in the existing body of scientific literature and proposes the adoption of “Thyroid Eye Disease” (TED) as standardized terminology for the inflammatory disease of the eye and orbit associated with thyroid autoimmunity.

## Methods

To determine the terminology use in the scientific literature for the inflammatory disease of the eye and orbit associated with thyroid autoimmunity, we searched all 2000, 2010 and 2020 publications indexed in the PubMed database for 6 keywords. The keywords were: Thyroid eye disease, thyroid ophthalmopathy, thyroid orbitopathy, Graves or Graves’ ophthalmopathy, Graves or Graves’ orbitopathy, and Graves’ eye disease. The search was performed with temporal limits and two 10-year intervals in order to generate a trend for the utilization of these terms. In addition to the predominant terminology used in the title and abstract, we then recorded authors’ characteristics including primary specialty, as well as the composition of the team as single specialty or multidisciplinary. We also recorded journal specialty and article type. Articles where these variables could not be determined were excluded.

We then also reviewed the terminology used by the professional societies that have a dedicated interest in this field (AAO, ATA, EUGOGO, ETA, ITEDS), and identified the terms that they have used in their official statements or guidelines over the same period.

IRB review was not needed since no protected health information was involved in this study.

## Results

**Figure 1:**
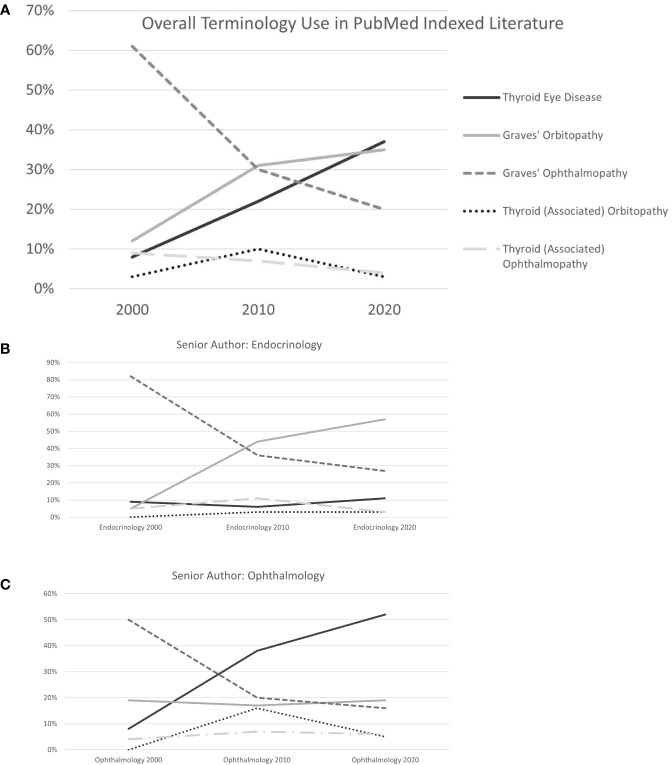
**(A)** Terminology Use Overall. **(B)** Terminology Use by Endocrinologists. **(C)** Terminology Use by Ophthalmologists.

- Terminology trends in the literature from 2000 to 2020:

The overall number of search results included in analysis increased from 77 in 2000 to 299 in 2020 (**Figure 1**), an increase of 388%. The 3 most commonly used terms in the 2000 literature were Graves’ Ophthalmopathy (61%), Graves’ Orbitopathy (12%) and Thyroid- (Associated) Ophthalmopathy (9%). This changed in 2010 to Graves’ Orbitopathy (31%), Graves’ Ophthalmopathy (30%) and Thyroid Eye Disease (22%). Popularity of the 3 most commonly used terms changed again from 2010 to 2020, when the predominantly used term was TED (37%), followed by Graves’ Orbitopathy (35%) and Graves’ Ophthalmopathy (20%).

- Impact of author specialty and journal-intended audience:

In endocrinology journals, Graves’ Ophthalmopathy was the predominant term in 2000 (84%), taken over by Graves’ Orbitopathy in 2010 (47%) and 2020 (67%) as shown in **Table 1**. Use of Graves’ Ophthalmopathy decreased at those 2 more recent time points (32% and 18%), and TED was used infrequently in 2000, 2010 and 2020 (8%, 2.6% and 12%, respectively). Similarly, the most commonly used term in ophthalmology journals in 2000 was Graves’ Ophthalmopathy (46%). In 2010 and 2020, this changed to TED (30% and 57%), followed by Graves’ Orbitopathy (25% and 16%) and Graves’ Ophthalmopathy (23% and 16%). In publications with endocrinologist and ophthalmologist senior authors, respective terminology trends were similar to patterns in journals of those specialties (**Table 1** and **Figures 1B, C**). Publications by multidisciplinary author teams showed a steady increase in use of TED from 2000 to 2010 and 2020 (9% to 23% to 32%), an initial large drop with recent stabilization for Graves’ Ophthalmopathy (64% to 21% to 25%) and predominant but in the recent decade decreasing use of Graves’ Orbitopathy (11% to 42% to 38%).

- Changes in terminology used by professional societies:

**Table 1:** 

Journal or Senior Author (SA) specialty	Thyroid Eye Disease	Graves’ orbitopathy	Graves’ ophthalmopathy	Thyroid-(associated) orbitopathy	Thyroid-(associated) ophthalmopathy	Other	Total(2000: 77)(2010: 134)(2020: 299)
Endocrinology Journals 2000	2 8%	–	21 84%	–	1 4%	1	25 (32%)
Endocrinology Journals 2010	1 2.6%	18 47%	12 32%	3 8%	4 11%	–	38 (27%)
Endocrinology Journals 2020	9 12%	52 67%	14 18%	3 4%		–	78 (26%)
Ophthalmology Journals 2000	2 7%	3 11%	13 46%	1 4%	4 14%	4	28 (36%)
Ophthalmology Journals 2010	21 30%	17 25%	16 23%	7 10%	8 12%	–	69 (50%)
Ophthalmology Journals 2020	77 57%	22 16%	22 16%	6 4%	8 6%	1	136 (45%)
Endocrinology SA 2000	2 9%	1 5%	18 82%	–	1 5%	–	22 (29%)
Endocrinology SA 2010	2 6%	16 44%	13 36%	1 3%	4 11%	–	36
Endocrinology SA 2020	9 11%	45 57%	21 27%	2 3%	2 3%	–	79
Ophthalmology SA 2000	2 8%	5 19%	13 50%	–	1 4%	4	26 (34%)
Ophthalmology SA 2010	23 38%	12 17%	12 20%	10 16%	4 7%	–	61
Ophthalmology SA 2020	81 52%	30 19%	25 16%	7 5%	9 6%	–	155

To determine consensus trends, we studied terminology use in publications and on websites of 5 large professional societies: AAO, ATA, EUGOGO, ETA, ITEDS. Detailed data are displayed in **Supplementary Table 1**. In the most recently published statements and guidelines, 4 out of the 5 societies used TED, while EUGOGO used Graves’ Orbitopathy. The AAO, ATA and ETA used Graves’ orbitopathy and Graves ophthalmopathy in earlier publications. In some cases, multiple terms are used in the same paragraph on current websites, which underscores the lack of consistency even within a single professional organization.

## Discussion

Our review of the literature shows that there is a trend for increasing use of TED by medical societies and ophthalmologists, while Graves’ orbitopathy remains the most likely used term by endocrinologists. Medical terminology is determined by convention and practice guideline recommendations, and may be modified based on new research findings, better understanding of the disease process, or to improve communication between specialties and with patients.

### Why Graves orbitopathy or ophthalmopathy is a problematic term:

The inflammatory disease of the eye and orbit associated with thyroid autoimmunity is a complex condition that mainly affects orbital tissues including extraocular muscles and fat, but can also cause pathology of eyelids, conjunctiva, cornea, optic nerve and retina (4–6). While most cases occur in the setting of the autoimmune hyperthyroidism caused by Graves’ disease, about 10% of patients will not be hyperthyroid at the time of the ophthalmic diagnosis (7), either because of sequential onset of eye and thyroid involvement, complete lack of functional thyroid abnormalities or in patients who develop eye disease associated with autoimmune hypothyroidism (8). In contrast to this reality, the eponym “Graves” as part of terminology for eye disease implies a hyperthyroid state, as the defining characteristic of Graves’ disease. Studies have shown confusion among patients regarding the possibility of eye disease occurring without hyperthyroidism (9). The authors have experienced similar variability in medical knowledge among referring providers in their clinical practice. Many patients referred to our multidisciplinary TED clinic have experienced a delay in diagnosis, because it was felt that normal thyroid hormone levels and TSH can rule out active eye disease. Some patients were calling their eye disease Graves’ disease, and thus opening the door for miscommunication when discussing prior evaluation and treatment geared towards thyroid or eye pathology.

A recently published correspondence describes the great variation of nomenclature that can be found even within a single journal issue, and makes a recommendation based on the authors’ opinion regarding scientific and clinical accuracy (10). While the existing conundrum is adequately described, the study was limited to reporting the respective number of search results for 11 keyword synonyms in different electronic databases, but did not further analyze trends over time, or association with author and journal specific factors. Our review of TED related literature from 2000, 2010 and 2020 showed that TED narrowly surpassed Graves’ orbitopathy as the most used term in more recent publications (**Figure 1**). Use of “Graves Ophthalmopathy”, as well as other less common terms including “thyroid-(associated) orbitopathy” and “thyroid- (associated) ophthalmopathy”, decreased during this 10-year period. While articles using TED and Graves’ orbitopathy together made up 20% in 2000, this number increased to 53% in 2010 and 72% in 2020. These findings indicate the ability of the medical and scientific community to change predominant terminology use over a relatively short period, as well as a general trend towards standardization. There is a clear specialty-dependent preference between those two most used terms: Endocrinology journals and senior authors mainly use Graves’ orbitopathy, while ophthalmology journals and senior authors prefer TED. This may reflect familiarity with the broader manifestations that ophthalmologists treat in patients with TED, aside from inflammation of orbital tissues. The term “Graves’ orbitopathy” is integrated in the name of the European Group on Graves’ Orbitopathy (EUGOGO), who has led several landmark clinical trials and developed widely accepted practice guidelines. However, on their website, the term TED is introduced immediately following GO, and multidisciplinary centers of excellence are described as “Combined Thyroid Eye Clinics (11).

The current real-world usage of different terms outside of scientific literature, which reflects what patients and other lay people encounter when researching periocular inflammatory disease associated with thyroid autoimmune disease, can be estimated by completing a Google search: The term “thyroid eye disease” yields over 83 million results, compared to 323,000 for Graves’ ophthalmopathy and only 124,000 for Graves’ orbitopathy (accessed from a U.S. server, Sep 12, 2022).

## Conclusion

We propose Thyroid Eye Disease (TED) as the preferred term for eye and periocular inflammatory disease associated with thyroid autoimmune disease. We base this recommendation on the increasing use by ophthalmologists as well as endocrinologists, and the better encompassing of the associated clinicopathologic processes. TED can be understood much more easily by patients, and for clinicians it eliminates the confusion about hyperthyroid state as a diagnostic criterium. Terminology trends in multidisciplinary publications and professional societies support this recommendation and highlight potential beneficial effects for collaborative care.

## Data availability statement

The original contributions presented in the study are included in the article/**Supplementary Material**. Further inquiries can be directed to the corresponding author.

## Author contributions

LW (first and corresponding author) - drafting the manuscript, generating analysis, and creating final manuscript. MS (senior author) - generating hypothesis, support in analysis and critical review of manuscript. EB and AT - critical review of the manuscript. YR – data collection, analysis, and review of manuscript. KR – analysis and review of manuscript. All authors contributed to the article and approved the submitted version.

## Conflict of interest

The authors declare that the research was conducted in the absence of any commercial or financial relationships that could be construed as a potential conflict of interest.

## Publisher’s note

All claims expressed in this article are solely those of the authors and do not necessarily represent those of their affiliated organizations, or those of the publisher, the editors and the reviewers. Any product that may be evaluated in this article, or claim that may be made by its manufacturer, is not guaranteed or endorsed by the publisher.
