# Supplementary material for: Thyroid eye disease or Graves’ orbitopathy: What name to use, and why it matters
Source: Front Endocrinol (Lausanne). 2022 Nov 28;13:1083886. doi: 10.3389/fendo.2022.1083886 (PMC9742525; doi:10.3389/fendo.2022.1083886)
Supplement: Supplementary file 1 [file Table_1.docx]

Supplementary Table 1. Terminology use in publications and websites of large professional societies.

| ***Organization*** | ***Terminology (Article and date)*** | ***Terminology (Article and date)*** | ***Terminology (Article and date)*** | ***Presented at*** | ***WEBSITE*** |
| --- | --- | --- | --- | --- | --- |
| **ATA** | Graves ophthalmopathy - **GO** (2011 Hyperthyroidism guidelines) | Graves orbitopathy - **GO** (2016 Hyperthyroidism guidelines) | Thyroid eye disease - **TED** (2022 TED consensus statement) | American Thyroid Association Annual Meeting; Sept. 30-Oct. 3, 2021 (virtual meeting). | Thyroid eye disease, Graves' eye disease Graves ophthalmopathy  and Graves orbitopathy (patient education section) |
| **ETA** |  | Graves orbitopathy - **GO** (2018 Hyperthyroidism guidelines ) | Thyroid eye disease - **TED** (2022 TED consensus statement) | American Thyroid Association Annual Meeting; Sept. 30-Oct. 3, 2021 (virtual meeting). | No information on eye disease |
| **EUGOGO** | Graves' orbitopathy- **GO** (2003 Multi-center study on characteristics and treatment strategies) | Graves' orbitopathy- **GO** (2017 Position Statement) |  |  | Graves' orbitopathy- GO (title page); mentions "Thyroid Eye Disease"  and "Dysthyroid Orbitopathy" as alternative terms |
| **ITEDS** | Thyroid eye disease - **TED** (2009 Development of criteria for evaluating clinical response) | Thyroid eye disease - **TED** (2018 Soft Tissue Metrics in Thyroid Eye Disease) |  |  | Thyroid eye disease- TED (title page) |
| **AAO** | Graves ophthalmopathy - **GO** (2008 Orbital radiation for graves ophthalmopathy) | Thyroid eye disease - **TED** (2022 Orbital Radiation for Thyroid Eye Disease) |  |  | Thyroid eye disease- TED (AAO Education Center) |
